# Diagnostic utility of snail in metaplastic breast carcinoma

**DOI:** 10.1186/1746-1596-5-76

**Published:** 2010-11-26

**Authors:** Aziza Nassar, Nicole Sookhan, Marta Santisteban, Sandra C Bryant, Judy C Boughey, Tamar Giorgadze, Amy Degnim

**Affiliations:** 1 Department of Laboratory Medicine and Pathology, Mayo Clinic, Rochester, MN, USA; 2 The Breast and Oncology Center, St. Mary's Health System, Southbury, CT, USA; 3 Department of Medical Oncology, Clínica Universidad de Navarra, Spain; 4 Department of Health Sciences Research, Mayo Clinic, Rochester, MN, USA; 5 Department of Surgery, Mayo Clinic, Rochester, MN, USA; 6 Department of Pathology, Henry Ford Hospital, Detroit, MI, USA

## Abstract

Metaplastic breast carcinoma (MBC) is a rare subtype of breast cancer characterized by coexistence of carcinomatous and sarcomatous components. Snail is a nuclear transcription factor incriminated in the transition of epithelial to mesenchymal differentiation of breast cancer. Aberrant Snail expression results in lost expression of the cell adhesion molecule E-cadherin, an event associated with changes in epithelial architecture and invasive growth. We aimed to identify the utility of Snail, and of traditional immunohistochemical markers, in accurate MBC classification and to evaluate clinicopathologic characteristics and outcome.

We retrospectively reviewed 34 MBC cases from January 1997 to September 2007. The control group contained 26 spindle cell lesions. Immunohistochemistry used Snail, p63, epidermal growth factor receptor (EGFR), OSCAR, and wide spectrum cytokeratin (WS-KER). Negative was a score less than 1%. We found that Snail and EGFR are sensitive (100%) markers with low specificity (3.8% and 19.2%) for detecting MBC. p63 and WS-KER are specific (100%), with moderate sensitivity (67.6% and 76.5%); OSCAR is sensitive (85.3%) and specific (92.3%). A combination of any 2 of the p63, OSCAR, and WS-KER markers increased sensitivity and specificity. MBCs tended to be high-grade (77%), triple negative (negative for estrogen receptor, progesterone receptor, and *HER2*) [27/33; 81.8%], and carcinomas with low incidence of axillary lymph node involvement (15%), and decreased disease-free [71% (95%CI: 54%, 94%) at 3 yrs.) and overall survival. A combination of p63, OSCAR and WS-KER are useful in its work-up. On the other hand, Snail is neither a diagnostic nor a prognostic marker for MBC.

## Background

Metaplastic breast carcinoma (MBC) is a rare subtype of breast cancer characterized by carcinomatous and sarcomatous components. Clinically, MBCs have a large size at diagnosis, lack expression of hormone receptors, and have a lower incidence of regional lymph nodes and a higher rate of systemic disease than ductal carcinomas of the breast [1]. Thus, the likelihood for recurrence of MBC is high, translating into a poor outcome. MBCs comprise less than 5% of mammary adenocarcinomas, and they generally present as rapidly growing, palpable tumors with circumscribed contours and a high-density mass with associated architectural distortion radiographically [1-3].

Customarily, MBCs are divided into 2 main categories: squamous and heterologous or pseudosarcomatous metaplasia. They have lower frequency of axillary lymph node metastases than non-metaplastic high-grade carcinomas [1]. Prognosis of MBC is determined by stage at diagnosis. It is unclear whether the histologic type of metaplasia has a significant effect on prognosis.

Transformation of the carcinomatous component into the sarcomatous component through epithelial-to-mesenchymal transition could explain the origin of the MBC [4]. This transition is a physiologic program used in embryogenesis and also activated during cancer invasion, progression and metastasis, in which cancer cells lose their adherent and polarity features and change into a mesenchymal phenotype with a more elongated cellular shape for increasing motility.

Association of breast cancer and epithelial-to-mesenchymal transition has been described in the medical literature [5], and now its role in the generation of the breast cancer stem cell phenotype has acquired a heightened interest [6,7]. Indeed, the tumoral microenvironment enhances extracellular stimuli such as increased matrix metalloproteinases production, to facilitate migration and invasion.

Epithelial-to-mesenchymal transition has been related to upregulation of the transcriptional repressor Snail [8], which is associated with loss of the epithelial adhesion molecule E-cadherin [9,10], and predicts a worse outcome in progression-free survival for women with breast cancer [8]. High Snail expression in breast cancers found with microarray analysis was significantly associated with a poor relapse-free survival in nonmetaplastic breast carcinomas [11-13]. Moreover, Snail expression predicts disease-free survival independently of lymph node status and tumor size [8]. A negative correlation was shown between Snail and estrogen receptor expression driven by the MTA3 (metastasis-associated protein) pathway [14].

Accurate diagnosis and differentiation of MBC from other spindle cell lesions of the breast can be challenging, especially in core needle biopsies [15]. In the present study, our primary aim was to study and compare Snail with other known traditional immunomarkers used in identifying MBC and to evaluate its correlation with tumor characteristics and outcome. Our secondary aim was to review our experience in the multidisciplinary management of MBC cases over a 10-year period, examining clinicopathologic features, treatment, and outcomes.

## Materials and methods

### Tissue specimens

The study was approved by the institutional review boards of our respective Institution. We conducted a retrospective review of 34 patients who received a diagnosis of MBC at a tertiary referral center from February 1997 to August 2007. Patient age, tumor size and grade, nodal status, hormone receptor status, initial treatment, recurrence, and follow up information were reviewed (Table 1).

**Table 1:** Clinicopathologic factors of 34 patients with metaplastic breast carcinoma

**Age (years)**, mean (range)	61.8 (32-90)	
**Histologic grade**, n (%)		
Low grade	6 (17.6%)	
Intermediate grade	2 (5.9%)	
High grade	26 (76.5%)	

**Histologic subtypes**		
Spindle cell	20 (58.8%)	
Mixed squamous and spindle cell	5 (14.7%)	
Squamous	4 (11.8%)	
Matrix producing	3 (8.8%)	
Adenosquamous	2 (5.9%)	

**Tumor diameter (cm)**, median (range)	3.0 (0.8-24)	

**Tumor size**	T1	10 (29.4%)
	T2	14 (41.2%)
	T3	6 (17.6%)
	T4	4 (11.8%)

**Receptor Status**		
ER positive	4 (12.1%)	
PR positive	3 (9.1%)	
Her2 positive	2 (5.9%)	

**Lymph node status**		
Positive	5 (14.7%)	
Negative	29 (85.3%)	

**Surgery**		
Mastectomy	21 (61.8%)	
BCT	13 (38.2%)	

**Adjuvant treatment**		
Chemotherapy	14 (41.2%)	
Radiation	17 (50.0%)	

**Recurrence**	Local	3 (8.8%)
	Distant	4 (11.8%)

**Overall survival**, median (95% CI*)		
1 year	82.8% (70.1%, 97.8%)	
3 years	58.4% (42.0%, 81.4%)	

**Disease-free survival**, median (95% CI*)		
1 year	78.5% (64.4%, 95.5%)	
3 years	71.3% (54.4%, 93.6%)	

*CI = Confidence Interval

The cases of MBC contained 20 cases of spindle cell type, 5 mixed spindle and squamous cell type, 4 squamous cell type, 3 matrix producing type, and 2 low-grade adenosquamous carcinoma. All patients with MBC were females with an average age of 62 yrs. (range: 32 - 90 yrs.). All tumors were characterized histologically according to Wartgotz's criteria [16-19]. The control group consisted of 26 spindle cell lesions: 14 phyllodes tumors, 8 pseudoangiomatous stromal hyperplasias (PASH), and 4 myofibroblastomas. The majority of the patients in the control group were females (23) with only few males (3) who presented with myofibroblastoma. The average age in the control group is 53 yrs. (range: 17 - 86 yrs.).

### Immunohistochemistry

The following immunohistochemical stains were performed: Snail, epidermal growth factor receptor (EGFR), OSCAR (a broad spectrum cytokeratin), wide spectrum cytokeratin (WS-KER) and p63, using these antibodies and positive controls listed in Table 2. Negative controls were run simultaneously and had primary antibody replaced with buffer. EGFR immunostain was performed with kits approved by the Food and Drug Administration (EGFR PharmDX;, Dako North America, Inc, Carpinteria, California) used in accordance with manufacturer instructions for antigen retrieval and immunostaining method. Antigen retrieval for all other antibodies was conducted in citrate buffer (pH, 6.0) under a pressure of 15 pounds per square inch for 3 minutes. The EnVision+ Dual Link Kit (Dako) was used for all immunostaining, with an automated slide stainer and with diaminobenzidine as chromogen and hematoxylin as counterstain.

**Table 2:** Immunohistochemical stains used, including their clones, dilutions, source and controls

Antibody	Monoclonal/polyclonal	Clone	Dilution	Source	Positive Control
SNAIL	M	AbcamAb17732	1:500	DAKOCarpinteria, CA	Breast cancer

EGFR	M	DAKO kit	prediluted	DAKOCarpinteria, CA	Colon cancer

OSCAR	M	OSCAR	1:200	DAKOCarpinteria, CA	Tonsil

WS-KER	P	Cytokeratin Wide Spectrum (WSS)	1:1600	DAKOCarpinteria, CA	Breastcancer

P63	M	PIN2 cocktail	prediluted	DAKOCarpinteria, CA	Prostate cancer

EGFR = epidermal growth factor receptor; WS-KER = wide spectrum keratin

Immunohistochemical staining was performed as follows. Formalin-fixed, paraffin-embedded samples, cut at 5 μm onto charged slides and baked at 60°C for 40 minutes prior before staining, were deparaffinized with 3 changes of xylene and rehydrated in a series of graded alcohols (100% ethanol, 95% ethanol, and 70% ethanol), then rinsed well in running distilled water. Slides were placed in a preheated citrate retrieval buffer (pH, 6.0) for 30 minutes, in a water steamer, cooled in the buffer for 5 minutes, and rinsed for 5 minutes in running distilled water.

Slides were placed on an automated slide stainer (AS100 Autostainer Plus; DAKO, Carpinteria, CA) for the following procedure (at room temperature). Sections were incubated with 3% hydrogen peroxide in ethanol for 5 minutes to inactivate endogenous peroxidases. They then were incubated in primary antibody for 30 minutes, rinsed with TBST wash buffer (S3006, Tris-Buffered Saline with Tween 20; DAKO) and incubated for 15 minutes with a peroxidase-labeled polymer conjugated to the secondary antibody. After a rinse with TBST wash buffer, sections were incubated in 3,3'-diaminobenzidine (K3468, DAB+ Substrate Chromogen; Dako) for 5 minutes, counterstained with modified Schmidt's hematoxylin for 5 minutes, followed by a 3- minute tap water rinse to set counterstain, dehydrated through graded alcohols (70% ethanol, 95% ethanol, and 100% ethanol), cleared in 3 changes of xylene, and mounted with permanent mounting media.

Antibody staining was membranous for EGFR, nuclear for PIN2 cocktail (p63) and Snail, and cytoplasmic for OSCAR and WS-KER. For all markers, scoring was performed using a score of less than 1% as negative. Several different microscopic fields (at least 10) per low and medium-power fields are examined for staining assessment.

### Statistical Analysis

Results were analyzed statistically with the sensitivity, specificity, and positive and negative predictive values for the different antibodies studied. Disease-free survival and overall survival were analyzed by the Kaplan-Meier survival curves. Associations of overall survival and surgery type, chemotherapy and radiotherapy were performed using the log rank test. Associations of tumor characteristics and snail percentage were assessed using Wilcoxon rank sum tests, while the association between snail percentage and age or tumor size was assessed using linear regression. In all cases, significance was defined as p-value < 0.05.

## Results

### Clinicopathologic characteristics (Table 2)

A total of 34 patients were identified (mean age, 61.8 y [range, 32-90 y]). Of them, 29 patients (85%) presented with a palpable lump and 5 (15%) had an abnormal mammogram; 27 (81.8%) patients had triple-negative hormonal receptor status (negative for estrogen receptor, progesterone receptor, and *HER2*). Approximately 76% (26/34) of patients have high-grade tumors. The distribution of tumor sizes was 10 patients with T1, and 14, 6 and 4 patients with T2, T3 and T4 tumors respectively.

**Figure 1:**
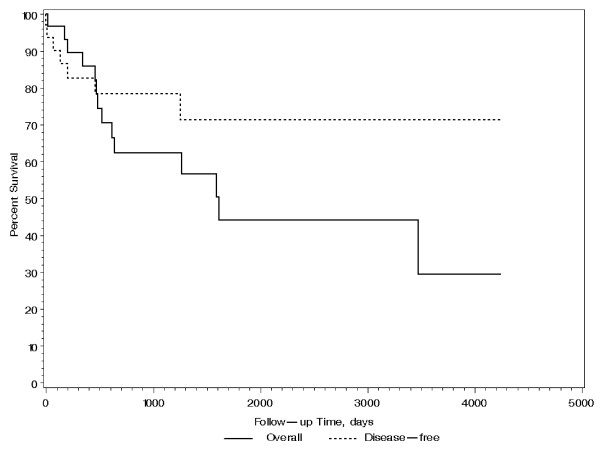
**Overall and disease-free survival of patients with MBC**.

**Figure 2:**
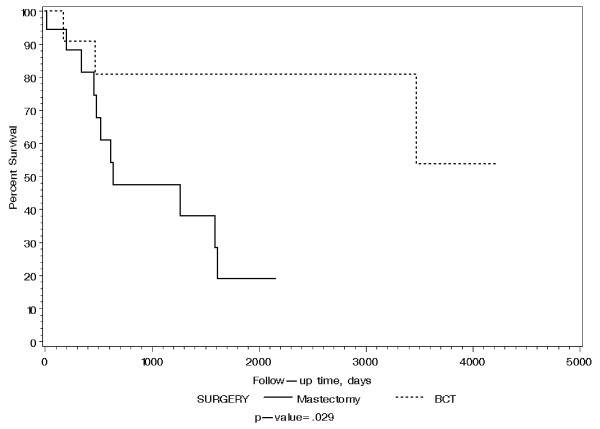
**Survival of patients with BCT and mastectomy for treatment of MBC**.

All patients had sentinel lymph node biopsy (100%), and only four patients had axillary lymph node (ALN) dissection (30.8%; 4/13). Five (15%) patients were lymph node positive. Those with positive ALNs have larger (pT2 or above) tumor size (p = 0.02). Twenty-one (62%) patients underwent mastectomy and 13 (38%) underwent breast conservation therapy (BCT). Fourteen patients (41%) received chemotherapy and seventeen (50%) patients received radiotherapy, 13 of whom received breast radiation as part of BCT. Neither chemotherapy nor radiotherapy increased survival of these patients (p = 0.87 and 0.13, respectively). Two patients were lost to follow up, with a median follow-up time of 21 months for the remaining 32. Three patients had local recurrences which occurred within 6 months, and 4 had distant metastases to lung and bone. Five patients died within the first year of diagnosis, 9 additional patients died by 5 years, and 2 more died by 10 years. The Kaplan-Meier estimated disease-free survival and 95% confidence interval (CI) was found to be 78.5% (64.4%, 95.5%) at 1 year and 71.3% (54.4%, 93.6%) at 3 years. The Kaplan-Meier estimated overall survival and 95% CI was found to be 82.8% (70.1%, 97.8%) at 1 year and 58.4% (42.0%, 81.4%) at 3 years as shown in Figure 1. Patients treated with BCT had statistically better overall survival than those with mastectomy (p = 0.029 by log rank test) as shown in Figure 2. This is partly attributed to lower tumor size (pT) in patients who underwent BCT (median tumor size is 1.5 cm) than those who had mastectomy (median tumor side is 4.8 cm) [p < 0.0001]. Although not statistically significant (p = 0.828), mastectomy patients more frequently had high grade tumors (81%) compared with BCT patients (69%). Finally, survival of these patients was not associated with age, tumor size, grade, ALN status, hormone receptor and HER2 status, and type of treatment (all p > 0.06).

### Immunohistochemical analysis

**Figure 3:**
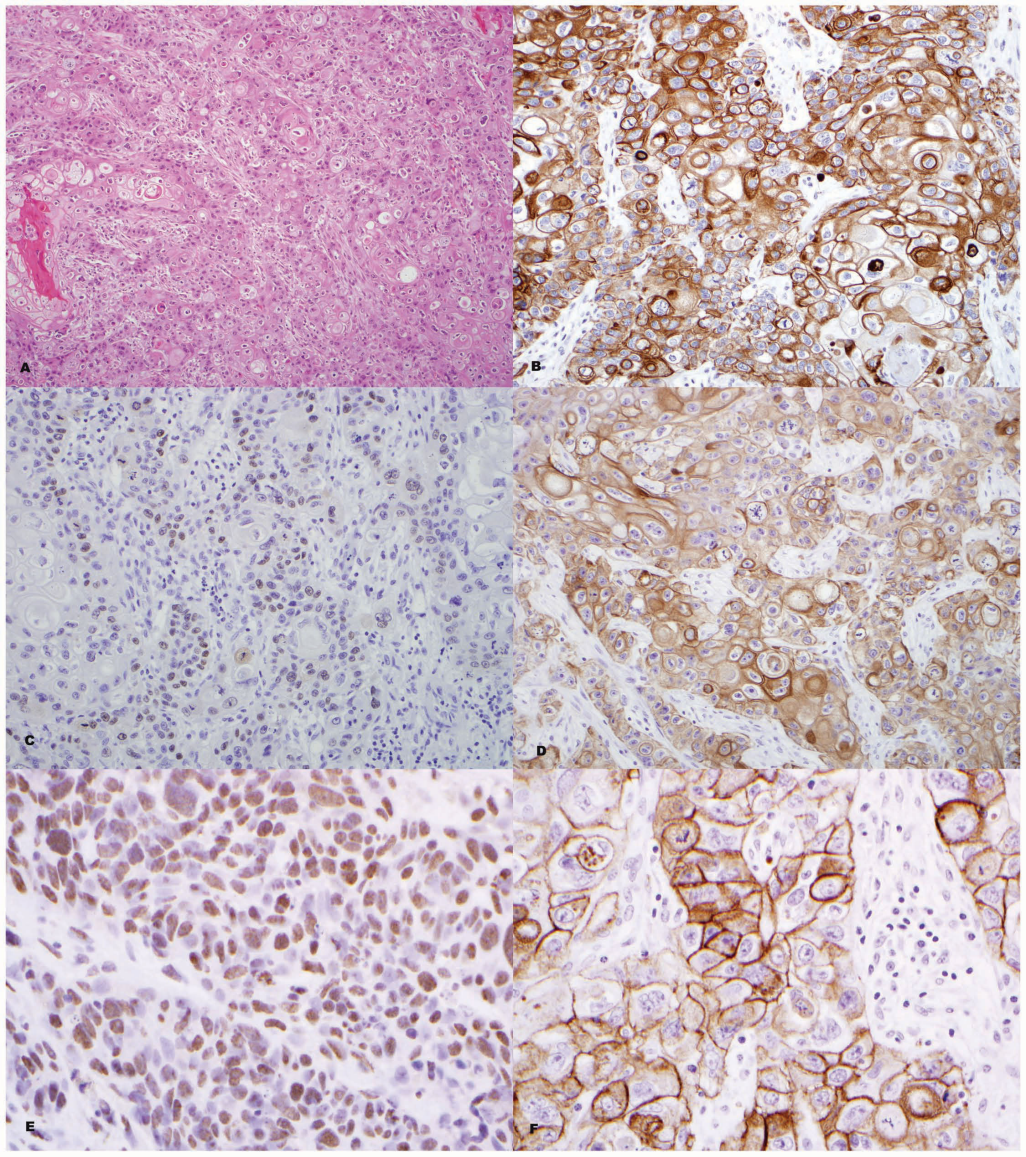
**High grade squamous cell carcinoma (A; H&E - 40×) with positive staining for OSCAR (B; 40×), p63 (C; 40×), WS-KER (D; 40×), SNAIL (E; 60×) and EGFR (F; 60×)**.

**Figure 4:**
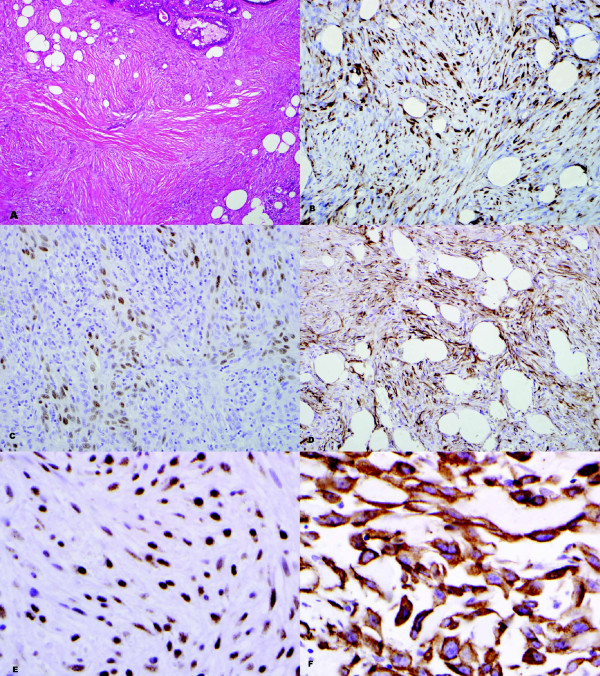
**High grade spindle carcinoma (A; H&E - 40×) with positive staining for OSCAR (B; 40×), p63 (C; 40×), WS-KER (D; 40×), SNAIL (E; 60×) and EGFR (F; 60×)**.

The results of the immunohistochemical analyses are as shown in Tables 3 &4 and Figures 3 &4. Snail was a sensitive (100%) marker for identifying MBC. However it was not a specific (3.8%) marker, as it was seen in other spindle cell lesions, including myofibroblastoma (4/4; 100%); phyllodes tumor (14/14; 100%) and PASH (7/8; 87.5%).

**Table 3:** Immunoexpression of the different markers in MBC, myofibroblastomas phyllodes tumor, and PASH

Antibody	MBC (%)	Myofibroblastoma (%)	Phyllodes Tumor (%)	PASH (%)
***SNAIL***	34/34 (100%)	4/4 (100%)	14/14 (100%)	7/8 (87.5%)
***EGFR***	34/34 (100%)	0/4 (0%)	13/14 (92.9%)	8/8 (100%)
***OSCAR***	29/34 (85.3%)	0/4 (0%)	2/14 (14.3%)	0/8 (0%)
***WS-KER***	26/34 (76.5%)	0/4 (0%)	0/14 (0%)	0/8 (0%)
***P63***	23/34 (56.7%)	0/4 (0%)	0/14 (0%)	0/8 (0%)

MBC = metaplastic breast carcinoma

PASH = pseudoangiomatous stromal hyperplasia

**Table 4:** Sensitivity, Specificity, Positive (PPV) and Negative (NPV) Predictive Values of the different immunomarkers (%)

Stains vs. Case	N	Overall Agreement, % (95% CI)	Sensitivity, % (95% CI)	Specificity, % (95% CI)	PPV	NPV
***EGFR ***	60	65.0 (52.4, 75.8)	100.0 (89.9, 100.0)	19.2 (8.5, 37.9)	61.8	100.0

***OSCAR***	60	88.3 (77.8, 94.2)	85.3 (69.9, 93.6)	92.3 (75.9, 97.9)	93.6	82.8

***P63***	60	81.7 (70.1, 89.4)	67.6 (50.8, 80.9)	100.0 (87.1, 100.0)	100.0	70.3

***SNAIL***	60	58.3 (45.7, 71.8)	100.0 (89.9, 100.0)	3.8 (0.7, 18.9)	57.6	100.0

***WSKER***	60	86.7 (75.8, 93.1)	76.5 (60.0, 87.6)	100.0 (87.1, 100.0)	100.0	76.5

***OSCAR & P63 & WSKER***	60	90.0 (79.9, 95.3)	88.2 (73.4, 95.3)	92.3 (75.9. 97.9)	93.8	85.7

***OSCAR & WSKER***	60	88.3 (77.8, 94.2)	85.3 (69.9, 93.5)	92.3 (75.9, 97.9)	93.6	82.8

***OSCAR & P63 ***	60	90.0 (79.9, 95.3)	88.2 (73.4, 95.3)	92.3 (75.9. 97.9)	93.8	85.7

***P63 & WSKER***	60	90.0 (79.9, 95.3)	82.4 (66.5, 91.7)	100.0 (87.1, 100.0)	100.0	81.2

EGFR = epidermal growth factor receptor; WS-KER = wide spectrum keratin

EGFR was also a sensitive marker (100%) but had a low specificity (19.2%), whereas p63 was a very specific marker (100%) for MBC but had a lower sensitivity (67.6%) compared to both EGFR and Snail. OSCAR keratin and WS-KER were both comparable in their sensitivity (85.3% and 76.5% respectively) and specificity (92.3% and 100% respectively) for detecting MBC. When several of those immunomarkers (OSCAR, p63 and WS-KER) were combined, both the sensitivity (82.4 to 88.2%) and specificity (92.3 to 100%) were increased, with the combination of p63 and WS-KER being the best two antibodies for detecting MBC in terms of high specificity (100%).

### Correlation of Snail with other tumor characteristics and survival

Snail expression (percentage of tumor cells staining) did not correlate with age, tumor size, grade, type of surgery (mastectomy vs. BCT), ALN status, hormone receptor and HER2 status, and type of treatment (hormonal therapy vs. chemotherapy vs. radiotherapy) (all p > 0.10). Furthermore, it did not correlate with survival (Hazard Ratio = 1.02; 95% CI: 0.98, 1.06; p = 0.45).

## Discussion

Our findings confirm findings from other studies reporting that the majority of MBC are high grade, hormone receptor negative and ALN negative. Despite presenting as node negative disease, the overall survival is low. We did not observe a significant difference with respect to overall survival between those who did and did not receive chemotherapy (p = 0.87 by log rank test), although the sample size is small. Similarly, no difference was seen between those who did and did not get radiotherapy (p = 0.13 by log rank test).

In one study, no differences were found in Snail expression as related to ductal or lobular cancer subtypes, tumor grade, or luminal versus basal array profile [8], but metaplastic carcinomas were not included in the analyses. In another study, Blanco et al. [20] did not find differences in Snail expression related to histologic type, lymph node disease and high-moderate tumor grade [20]. Similarly, we did not find any significant association between Snail expression and other clinicopathologic factors (age, tumor size, grade, type of surgery, ALN status, hormone receptor and HER2 status, and type of treatment).

Breast conservation appears to be a reasonable treatment option with better survival than mastectomy. Although most MBC are high grade, there are a few variants that are low grade and behave indolently, such as low grade adenosquamous [21] and fibromatosis-like spindle cell carcinoma [22,23]. Several investigators suggest that those variants of MBC can be treated with BCT if the tumor size allows [21,22]. Dave et al. [24] have found that breast conservation to be a reasonable treatment option with equivalent survival to mastectomy, and adjuvant radiation is essential for achieving high local control rates after conservative surgery [24]. Matrix-producing carcinoma and biphasic metaplastic sarcomatoid carcinoma (carcinosarcoma) are aggressive subtypes of MBC with a worse clinical outcome than conventional invasive ductal carcinoma (IDC) with a decreased locoregional recurrence-free survival (p = 0.001) and decreased distant recurrence-free survival (p = 0.001) [25]. Several investigators are suggesting modified radical mastectomy with adjuvant treatment (radiation and/or chemotherapy) for patients with aggressive subtypes of MBC, particularly for patients with T2 and higher stage disease [25,26]. Dave et al. [24] have shown BCT is equivalent to mastectomy in terms of survival. In our study, we found patients treated with BCT to have significantly better survival than those treated with mastectomy. Those patients treated with BCT presented with smaller tumor size and, generally, better tumor grade, indicating they may have had less severe disease.

Unlike our study and others [24,27], Khan et al. [28] and Sayed at al [29], have found higher rates of axillary lymph node involvement (40% and 53% respectively), and therefore proposed axillary lymph node staging. Furthermore, several investigators found that positive axillary nodes at presentation are strongly associated with worse survival [24,30]. In our study, only 4 of 13 (30.8%) patients have axillary lymph node involvement at presentation, although 21 did not have axillary nodes assessed.

Overall, MBC has a worse disease-free survival, and a significantly decreased overall survival, when compared to typical conventional breast carcinoma [1,28,29]. However, Gibson et al. [31] have found that survival in MBC appears to be similar to that of conventional ductal adenocarcinoma, when stratified by stage [31]. Furthermore, they did not find adjuvant chemotherapy to be of benefit, by multivariate analysis [31]. Using multivariate analysis, they also did not find an impact on recurrence or survival with regard to tumor size, age, menopausal status, nodal status, histologic subtype, adjuvant therapy, or extent of surgery [31]. In our study, we also found no difference in survival associated with age, tumor size, grade, ALN status, hormone receptor and HER2 status, or type of treatment, likely due to the small sample size providing limited statistical power.

MBC usually develop hematogenous metastases (lung and bone), in keeping with the sarcomatous phenotype [29,32]. Usually, MBC tends to be estrogen and progesterone receptor negative [28,29]. Metaplastic carcinomas are thought to be of basal-cell phenotype based on immunohistochemical profile [33], and therefore most likely will express basal keratins (CK5/6, CK14) [15]. A subset of MBC (specifically adenosquamous carcinoma) arises in association with papillomas or complex sclerosing lesions [34,35].

Since MBC mimic other spindle cell lesions of the breast, immunohistochemistry, specifically the use of cytokeratins, are valuable in differentiating between the two entities [22,23,27,36]. Carter et al. [27] found that pankeratin (MNF116) is the most sensitive marker (93%), followed by cytokeratins 14 (90%) for identifying MBC. Other markers that are positive in MBC but to a lesser extent include CAM 5.2 (40%) and AE1/3 (41%) [27]. Several recent studies have described that some metaplastic carcinomas exhibit myoepithelial differentiation. Dunne et al. [15] reported at least focal staining for smooth muscle actin (SMA) in 79% (11/14) of MBC. In addition, they noted frequent expression of the basal cell and myoepithelial keratins 34 βE12, CK5 and CK14 [15]. Reis-Filho et al. [37] also found frequent positivity for SMA and CK14, as well as immunoreactivity for S100 protein, p63, and the novel myoepithelial markers, maspin and p-cadherin. Other investigators have identified consistent expression of maspin and cadherins in sarcomatoid breast carcinoma [38]. Koker and Kleer [39] reported expression of p63 in all 10 spindle cell carcinomas examined, compared with 1 of 174 (0.6%) of nonmetaplastic breast carcinomas and 0 of 10 phyllodes tumors. In a study of 20 spindle cell metaplastic carcinomas, Leible et al. [40] found positive staining for p63 (70%), SMA (60%), S100 protein (45%) and CD10 (80%), in addition to frequent immunoreactivity for maspin, CD29, and 14-3σ (markers that appear to be preferentially expressed in myoepithelial cells). Tse et al. [41] found p63 to be a useful marker in the diagnosis of MBC with a sensitivity of 65%, specificity of 96%, a PPV and NPV of 96% and 66%, respectively, and an accuracy of 78%. In our study, we have found that OSCAR, WS-KER and p63 to be the most sensitive and specific markers for identifying MBC.

Although EGFR is expressed in MBC (66%), no EGFR or KIT activating mutations are present [33]. αB-crystallin is another novel marker found to be highly expressed in MBC (86%) [42]. The present study is the first to evaluate Snail as a possible marker for diagnosis of MBC. Although Snail is a sensitive marker (100%), it has a low specificity (3.8%) because it is expressed promiscuously in other spindle cells lesions of the breast.

## Conclusions

Snail is neither a good diagnostic nor a prognostic marker for MBC. MBC tend to be high grade, triple-negative (negative for estrogen and progesterone receptors and *HER2*) carcinomas with few axillary lymph node metastases and reduced overall survival. Our findings support the diagnostic utility of p63, OSCAR and WS-KER as a panel in the diagnosis of MBC and in the differentiation of these tumors from other spindle cell lesions of the breast.

## Abbreviations

EGFR: epidermal growth factor; MBC: metaplastic breast carcinoma; WS-KER: wide spectrum cytokeratin;

## Competing interests

The authors declare that they have no competing interests.

## Authors' contributions

AN: Concept and design of the study, data collection, data analysis and interpretation, writing of final draft and review of revised manuscript draft. NS: Data collection and analysis, revision and approval of final and revised manuscript draft. MS: Data analysis and interpretation, revision and approval of final and revised manuscript draft. SCB: Participated in the design of the study, performed statistical analysis and interpretation of data, revision and approval of final and revised manuscript draft. JCB: Critique and revision of final and revised manuscript draft. TC: Critique and revision of final and revised manuscript draft. AD: Critique and revision of final and revised manuscript draft. All authors read and approved the final and revised manuscript.

## References

[B1] Luini A Aguilar M Gatti G Fasani R Botteri E Brito JA Maisonneuve P Vento AR Viale G Metaplastic carcinoma of the breast, an unusual disease with worse prognosis: the experience of the European Institute of Oncology and review of the literature Breast Cancer Res Treat 2007 101 349 353 10.1007/s10549-006-9301-1 17009109

[B2] Günhan-Bilgen I Memis A Ustun EE Zekioglu O Ozdemir N Metaplastic carcinoma of the breast: clinical, mammographic, and sonographic findings with histopathologic correlation AJR 2002 178 1421 1425 12034610 10.2214/ajr.178.6.1781421

[B3] Park JM Han BK Moon WK Choe YH Ahn SH Gong G Metaplastic carcinoma of the breast: mammographic and sonographic findings J Clin Ultrasound 2000 28 179 186 10.1002/(SICI)1097-0096(200005)28:4<179::AID-JCU5>3.0.CO;2-Y 10751739

[B4] Lien HC Hsiao YH Lin YS Yao YT Juan HF Kuo WH Hung MC Chang KJ Hsieh FJ Molecular signatures of metaplastic carcinoma of the breast by large-scale transcriptional profiling: identification of genes potentially related to epithelial-mesenchymal transition Oncogene 2007 26 57 7859 71 10.1038/sj.onc.1210593 17603561

[B5] Knutson KL Lu H Stone B Reiman JM Behrens MD Prosperi CM Gad EA Smorlesi A Disis ML Immunoediting of cancers may lead to epithelial to mesenchymal transition J Immunol 2006 177 3 1526 33 16849459 10.4049/jimmunol.177.3.1526

[B6] Mani SA Guo W Liao MJ Eaton EN Ayyanan A Zhou AY Brooks M Reinhard F Zhang CC Shipitsin M Campbell LL Polyak K Brishen C Yang J Weinberg RA The epithelial-mesenchymal transition generates cells with properties of stem cells Cell 2008 133 4 704 15 10.1016/j.cell.2008.03.027 18485877 PMC2728032

[B7] Santisteban M Reiman JM Asiedu MK Behrens MD Nassar A Kalli KR Haluska P Ingle JN Hartmann LC Manjili MH Radisky DC Ferrone S Knutson KL Immune-induced epithelial to mesenchymal transition in vivo generates breast cancer stem cells Cancer Res 2009 69 7 2887 95 10.1158/0008-5472.CAN-08-3343 19276366 PMC2664865

[B8] Moody SE Perez D Pan TC Sarkisian CJ Portocarrero CP Sterner CJ Notorfrancesco KL Cardiff RD Chodosh LA The transcriptional repressor Snail promotes mammary tumor recurrence Cancer Cell 2005 8 3 197 209 10.1016/j.ccr.2005.07.009 16169465

[B9] Cano A Perez-Moreno MA Rodrigo I Locascio A Blanco MJ del Barrio MG Portillo F Nieto MA The transcription factor Snail controls epithelial-mesenchymal transitions by repressing E-cadherin expression Nat Cell Biol 2000 2 2 76 83 10.1038/35000025 10655586

[B10] Zhou BP Hung MC Wnt, hedgehog and Snail: sister pathways that control by GSK-3beta and beta-Trcp in the regulation of metastasis Cell Cycle 2005 4 6 772 6 10.4161/cc.4.6.1744 15917668

[B11] Sorlie T Perou CM Tibshirani R Aas T Geisler S Johnsen H Akslen LA Fluge O Pergamenschikov A Williams C Zhu SX Lǿnning PE Bǿrresen-Dale AL Brown PO Botstein D Gene expression patterns of breast carcinomas distinguish tumor subclasses with clinical implications Proc Natl Acad Sci USA 2001 98 19 10869 74 10.1073/pnas.191367098 11553815 PMC58566

[B12] van't Veer LJ Paik S Hayes DF Gene expression profiling of breast cancer: a new tumor marker J Clin Oncol 2005 23 8 1631 5 10.1200/JCO.2005.12.005 15755970

[B13] Wang Y Klijn JG Zhang Y Sieuwerts AM Look MP Yang F Talantov D Timmermans M Meijer-van Gelder ME Yu J Jutkoe T Berns EM Atkins D Foekens JA Gene-expression profiles to predict distant metastasis of lymph-node-negative primary breast cancer Lancet 2005 365 9460 671 9 15721472 10.1016/S0140-6736(05)17947-1

[B14] Fujita N Jaye DL Kajita M Geigerman C Moreno CS Wade PA MTA3, a Mi-2/NuRD complex subunit, regulates an invasive growth pathway in breast cancer Cell 2003 113 2 207 19 10.1016/S0092-8674(03)00234-4 12705869

[B15] Dunne B Lee AH Pinder SE Bell JA Ellis IO An immunohistochemical study of metaplastic spindle cell carcinoma, phyllodes tumor and fibromatosis of the breast Hum Pathol 2003 34 1009 1015 10.1053/S0046-8177(03)00414-3 14608534

[B16] Wargotz ES Deos PH Norris HJ Metaplastic carcinomas of the breast. II. Spindle cell carcinoma Hum Pathol 1989 20 732 740 10.1016/0046-8177(89)90065-8 2473024

[B17] Wargotz ES Norris HJ Metaplastic carcinomas of the breast. I. Matrix-producing carcinoma Hum Pathol 1989 20 628 635 10.1016/0046-8177(89)90149-4 2544506

[B18] Wargotz ES Norris HJ Metaplastic carcinomas of the breast. III. Carcinosarcoma Cancer 1989 64 1490 1499 10.1002/1097-0142(19891001)64:7<1490::AID-CNCR2820640722>3.0.CO;2-L 2776108

[B19] Wargotz ES Norris HJ Metaplastic carcinomas of the breast. IV. Squamous cell carcinoma of ductal origin Cancer 1990 65 272 276 10.1002/1097-0142(19900115)65:2<272::AID-CNCR2820650215>3.0.CO;2-6 2153044

[B20] Blanco MJ Moreno-Bueno G Sarrio D Locascio A Cano A Palacios J Nieto MA Correlation of Snail expression with histological grade and lymph node status in breast carcinomas Oncogene 2002 21 20 3241 6 10.1038/sj.onc.1205416 12082640

[B21] Rosen PP Ernsberger D Low-grade adenosquamous carcinoma. A variant of metaplastic mammary carcinoma Am J Surg Pathol 1987 11 351 358 10.1097/00000478-198705000-00003 3578645

[B22] Sneige N Yaziji H Mandavilli SR Perez ER Ordonez NG Gown AM Ayala A Low-grade (fibromatosis-like) spindle cell carcinoma of the breast Am J of Surg Pathol 2001 25 1009 1016 10.1097/00000478-200108000-00004 11474284

[B23] Brogi E Benign and malignant spindle cell lesions of the breast Semin Diagn Pathol 2004 21 57 64 10.1053/j.semdp.2003.10.007 15074560

[B24] Dave G Cosmatos H Do T Lodin K Varshney D Metaplastic carcinoma of the breast: a retrospective review Int J Radiat Oncol Biol Phys 2006 64 771 775 10.1016/j.ijrobp.2005.08.024 16246496

[B25] Downs-Kelly E Nayeemuddin KM Albarracin C Wu Y Hunt KK Gilcrease MZ Matrix-producing Carcinoma of the Breast: An Aggressive Subtype of Metaplastic Carcinoma Am J Surg Pathol 2009 33 4 534 41 10.1097/PAS.0b013e31818ab26e 19047898

[B26] Hennessy BT Giordano S Broglio K Duan Z Trent J Buchholz TA Babiera G Hortobagyi GN Valero V Biphasic metaplastic sarcomatoid carcinoma of the breast Ann Oncol 2006 17 605 613 10.1093/annonc/mdl006 16469754

[B27] Carter MR Hornick JL Lester S Fletcher CD Spindle cell (sarcomatoid) carcinoma of the breast: a clinicopathologic and immunohistochemical analysis of 29 cases Am J Surg Pathol 2006 30 300 309 16538049 10.1097/01.pas.0000184809.27735.a1

[B28] Khan HN Wyld L Dunne B Lee AH Pinder SE Evans AJ Robertson JF Spindle cell carcinoma of the breast: a case series of a rare histological subtype Eur J Surg Oncol 2003 29 600 603 10.1016/S0748-7983(03)00107-0 12943626

[B29] Al Sayed AD El Weshi AN Tulbah AM Rahal MM Ezzat AA Metaplastic carcinoma of the breast clinical presentation, treatment results and prognostic factors Acta Oncol (Stockholm, Sweden) 2006 45 188 195 10.1080/02841860500513235 16546865

[B30] Chao TC Wang CS Chen SC Chen MF Metaplastic carcinomas of the breast J Surg Oncol 1999 71 220 225 10.1002/(SICI)1096-9098(199908)71:4<220::AID-JSO3>3.0.CO;2-L 10440759

[B31] Gibson GR Qian D Ku JK Lai LL Metaplastic breast cancer: clinical features and outcomes Am Surg 2005 71 725 730 16468506

[B32] Kurian KM Al-Nafussi A Sarcomatoid/metaplastic carcinoma of the breast: a clinicopathological study of 12 cases Histopathology 2002 40 58 64 10.1046/j.1365-2559.2002.01319.x 11903598

[B33] Gilbert JA Goetz MP Reynolds CA Ingle JN Giordano KF Suman VJ Blair HE Jenkins RB Lingle WL Reinholz MM Adjei AA Ames MM Molecular analysis of metaplastic breast carcinoma: high EGFR copy number via aneusomy Mol Cancer Ther 2008 7 944 951 10.1158/1535-7163.MCT-07-0570 18413808 PMC2745608

[B34] Gobbi H Simpson JF Jensen RA Olson SJ Page DL Metaplastic spindle cell breast tumors arising within papillomas, complex sclerosing lesions, and nipple adenomas Mod Pathol 2003 16 893 901 10.1097/01.MP.0000085027.75201.B5 13679453

[B35] Denley H Pinder SE Tan PH Sim CS Brown R Barker T Gearty J Elston CW Ellis IO Metaplastic carcinoma of the breast arising within complex sclerosing lesion: a report of five cases Histopathology 2000 36 203 209 10.1046/j.1365-2559.2000.00849.x 10692021

[B36] Tse GM Tan PH Lui PC Putti TC Spindle cell lesions of the breast--the pathologic differential diagnosis Breast Cancer Res Treat 2008 109 199 207 10.1007/s10549-007-9652-2 17636400

[B37] Reis-Filho JS Milanezi F Paredes J Silva P Pereira EM Maeda SA de Carvalho LV Schmitt FC Novel and Classic Myoepithelial/Stem Cell Markers in Metaplastic Carcinomas of the Breast Appl Immunohistochem Mol Morphol 2003 11 1 8 10.1097/00022744-200303000-00001 12610349

[B38] Popnikolov NK Ayala AG Graves K Gatalica Z Benign Myoepithelial Tumors of the Breast Have Immunophenotype Characteristics Similar to Metaplastic Matrix Producing and Spindle Cell Carcinomas Am J Clin Pathol 2003 120 161 167 10.1309/G6CTR8MDTFUW19XV 12931544

[B39] Koker MM Kleer CG P63 expression in Breast Cancer. A Highly Sensitive and Specific Marker of Metaplastic Carcinoma Am J Surg Pathol 2004 28 1506 15122 10.1097/01.pas.0000138183.97366.fd 15489655

[B40] Leibl S Sommersacher A Denk H Moinfar F Metaplastic Breast Carcinomas: Are they of Myoepithelial Differentiation? Immunohistochemical Profile of the Sarcomatoid Subtype Using Novel Myoepithelial Markers Am J Surg Pathol 2005 29 347 353 10.1097/01.pas.0000152133.60278.d2 15725803

[B41] Tse GM Tan PH Chaiwun B Putti TC Lui PC Tsang AK Wong FC Lo AW p63 is useful in the diagnosis of mammary metaplastic carcinomas Pathology 2006 38 16 20 10.1080/00313020500444625 16484002

[B42] Sitterding SM Wiseman WR Schiller CL Luan C Chen F Moyano JV Watkin WG Wiley EL Cryns VL Diaz LK Alpha B-crystallin: a novel marker of invasive basal-like and metaplastic breast carcinomas Ann Diagn Pathol 2008 12 33 40 10.1016/j.anndiagpath.2007.02.004 18164413

